# Preservation of Pathological Specimens

**Published:** 1844-10-19

**Authors:** 


PRESERVATION OF PATHOLOGICAL SPECIMENS.

M. Pignè announces that a solution of creosote, in in the proportion of 4, 5, 6, 7, 8, or 10 drops, according to circumstances, to the litre, or pint and three quarters of water, forms an excellent, and of course very cheap, liquor for the preservation of specimens. An entire subject, or any portion of it, kept in the solution of 10 drops, preserves all its physical characters and properties unchanged for an indefinite length of time. Pathological specimens, that have been shrunk and blanched by twenty years' keeping in spirits, are speedily restored to their original form, size, and pliability, by being transferred to the creosote liquor. Portions of blood, pus, urine, &c., may be kept in it without undergoing any change, and examined at leisure.—*Lond. Med. Gazette.*

